# Altered protein phosphorylation as a resource for potential AD biomarkers

**DOI:** 10.1038/srep30319

**Published:** 2016-07-28

**Authors:** Ana Gabriela Henriques, Thorsten Müller, Joana Machado Oliveira, Marta Cova, Cristóvão B. da Cruz e Silva, Odete A. B. da Cruz e Silva

**Affiliations:** 1 Neuroscience and Signalling Laboratory, Department of Medical Sciences and Insitute of Biomedicine - iBiMED, University of Aveiro, 3810-193 Aveiro, Portugal; 2 Cell Signaling in Neurodegeneration (CSIN), Medical Proteome-Center, Ruhr-University Bochum, 44801 Bochum, Germany; 3 Laboratório de Instrumentação e Física Experimental de Partículas – LIP, Av. Elias Garcia 14 - 1°, 1000-149 Lisboa, Portugal

## Abstract

The amyloidogenic peptide, Aβ, provokes a series of events affecting distinct cellular pathways regulated by protein phosphorylation. Aβ inhibits protein phosphatases in a dose-dependent manner, thus it is expected that the phosphorylation state of specific proteins would be altered in response to Aβ. In fact several Alzheimer’s disease related proteins, such as APP and TAU, exhibit pathology associated hyperphosphorylated states. A systems biology approach was adopted and the phosphoproteome, of primary cortical neuronal cells exposed to Aβ, was evaluated. Phosphorylated proteins were recovered and those whose recovery increased or decreased, upon Aβ exposure across experimental sets, were identified. Significant differences were evident for 141 proteins and investigation of their interactors revealed key protein clusters responsive to Aβ treatment. Of these, 73 phosphorylated proteins increased and 68 decreased upon Aβ addition. These phosphorylated proteins represent an important resource of potential AD phospho biomarkers that should be further pursued.

The Aβ peptide, derived by proteolytic cleavage^1^,^2^ of the Alzheimer’s Amyloid Precursor Protein (APP), is associated with the onset of Alzheimer’s disease (AD). Aβ, typically around 40 amino acids long, is produced under basal conditions but in familial AD (Swedish mutation) the rate can increase by almost 10 fold^3^. Peptides range in length^4^,^5^; the longer species are more toxic. Aβ can be deposited as senile plaques (SPs) in AD patients’ brains and is generated via the endosomal/lysosomal degradation pathway, but its production can also be associated to the ER and Golgi/TGN^6^. However it is not solemnly a toxic peptide, as it appears to have important physiological functions. Aβ is present in the cerebral spinal fluid of AD patients but also of non-demented individuals and in media from neuronal cell cultures^7^,^8^,^9^. Further, Aβ appears to be involved in synaptic activity and protect against excessive glutamate release^10^,^11^, thus it is involved in excitability and neuronal survival. Other functions, include monitoring cholesterol transport^12^ and it may even have a role as a transcription factor^13^.

Protein phosphorylation is a key mechanism and regulates many cellular processes. Consequently abnormal protein phosphorylation has been linked to numerous human diseases including AD. Both APP and TAU phosphorylation have been associated with this dementia^14^,^15^. TAU, in a hyperphosphorylated state, forms neurofibrillary tangles (NFTs) which can deposit in the brain^16^. In AD other brain proteins such as neurofilaments, MAP1B, dynein, CRMP-2, β-tubulin and β-catenin are also hyperphosphorylated. Consistently, in AD, kinase activities and/or expression can be increased (GSK3β, CDK5, ERK1/2, JNK, p38MAPK). Likewise protein phosphatase (PP) activities and/or expression can be decreased (PP1, PP2, and PTEN - phosphatase and tensin homolog)^17^. Taken together, it is evident that protein (de)phosphorylation mechanisms are dysregulated in AD.

Aβ links many AD related anomalies. It can activate Src family protein kinases, activate phosphatidylinositol 3-kinase^18^ and the cAMP response element-binding protein phosphorylation^19^. Aβ mediated changes can result in phosphorylation of neuronal proteins, and contribute to the critical early AD pathogenic events, culminating in neuronal death and neurodegeneration^20^. Furthermore, Aβ is directly involved in stimulating kinases^21^,^22^,^23^ and inhibiting PP1 and PP2 activities, in a dose-dependent manner^24^. It is therefore not surprising that Aβ prompts the production of NFTs via mediating the expression levels and/or activities of TAU protein kinases and phosphatases^21^,^25^.

Clearly Aβ affects distinct cellular pathways many of which are regulated by reversible protein phosphorylation. In the work herein described primary neuronal culture lysates were collected upon Aβ exposure, enriched for phosphorylated proteins and the latter identified by mass spectrometry analysis. The approach revealed a series of phosphorylated proteins, whose levels were significantly altered upon Aβ exposure, across experimental sets. The proteins identified are strong biomarker candidates, and the networks presented reveal novel protein relationships and signalling cascades, relevant to unraveling the molecular basis of AD.

## Results

### Enrichment of the phosphoproteomes

**Figure 1:**
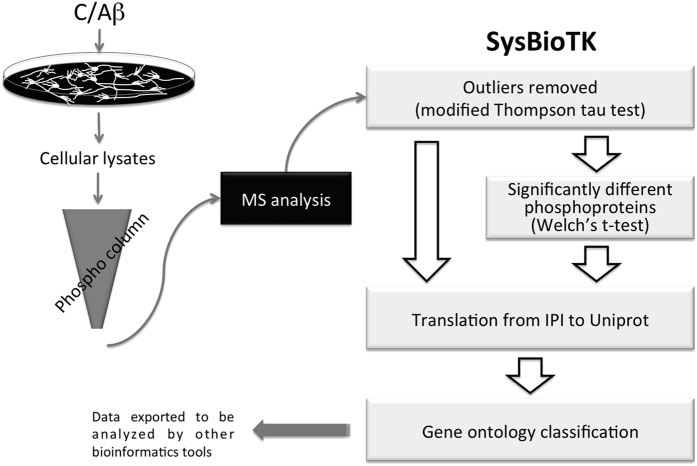
Workflow. The workflow describes sample processing and analysis. Primary rat neuronal cortical cultures were treated with Aβ, the lysates were collected and the phosphorylated proteins enriched in the phospho column. The eluted peptides were analyzed by mass spectrometry and the resulting data handled by the SysBioTK.

Primary neuronal cultures were exposed to Aβ and phosphorylated proteins enriched using the phospho column, were subjected to mass spectrometry analysis (Fig. 1). For all peptides obtained an accession IPI number was attributed. Although it was not the primary aim to identify specific phosphorylation sites, these were nonetheless readily detected. As examples, for the TAU protein (*Mapt* gene), three phosphorylated peptides (phosphopeptides) were identified (Supplementary Fig. S1) and one phosphorylated peptide was identified for GAPDH accession number IPI (Supplementary Fig. S2).

The data obtained from the mass spectrometry was subsequently analyzed using an informatics library (SysBioTK – Systems Biology Toolkit), specifically developed for this purpose (https://bitbucket.org/CrisXed/sysbiotk). An essential capacity of this platform is to translate accession number IPI to UniProt. The resulting accession numbers and corresponding gene lists were either handled by the SysBioTK (da Cruz e Silva, 2016 submitted), or submitted to other open access analysis tools.

Six experiments were carried out, to evaluate the phosphorylated proteins under basal conditions and upon Aβ exposure. As an initial step, all experimental datasets were analyzed for outliers by the modified Thompson tau, τ, test, (Fig. 1) and one of the experiments from the control condition was removed (Supplementary Table S1).

### Gene Ontology analysis

Under the experimental conditions implemented (Supplementary Table S1), 986 phosphoproteins were recovered following Aβ exposure (GpAβ–groupAβ) and 870 phosphoproteins under control conditions (GpC–groupControl).

**Figure 2:**
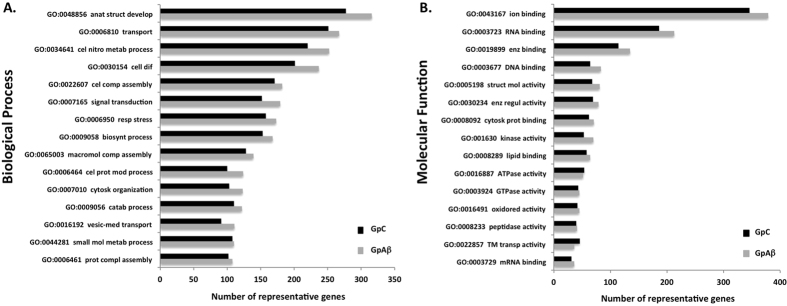
Gene Ontology of the phosphatomes. Genes encoding the eluted phosphorylated proteins under basal (GpC, black bars) conditions and upon Aβ addition (GpAβ, grey bars) were analyzed with respect to their Gene Ontology (GO) using the SysBioTK for Biological Process and Molecular Function. Abbreviations of GOs not in full: GO:0048856 anatomical structure development; GO:0034641 cellular nitrogen compound metabolic process; GO:0030154 cell differentiation; GO:0022607 cellular component assembly; GO:0007165 signal transduction; GO:0006950 response to stress; GO:0009058 biosynthetic process; GO:0065003 macromolecular complex assembly; GO:0006464 cellular protein modification process; GO:0007010 cytoskeleton organization; GO:0009056 catabolic process; GO:0016192 vesicle-mediated transport; GO:0044281 small molecule metabolic process; GO:0006461 protein complex assembly; GO:0019899 enzyme binding; GO:0005198 structural molecule activity; GO:0030234 enzyme regulator activity; GO:0008092 cytoskeletal protein binding; GO:0016491 oxidoreductase activity; GO:0022857 transmembrane transporter activity.

The GpC and GpAβ phosphatomes were analyzed with respect to Gene Ontology (GO) (Fig. 2). The distribution of the phosphorylated proteins across Molecular Functions and Biological Processes, for both GpAβ and GpC is similar. Top Biological Processes with the greater number of phosphorylated proteins are ‘anatomical structure development’, ‘transport’, ‘cellular nitrogen compound metabolic’, ‘cell differentiation’ and ‘signal transduction’. Top Molecular Functions identified are ‘ion binding’, ‘RNA binding’ and ‘enzyme binding’. There are no dramatic shifts or the appearance of novel Biological Processes or Molecular Functions upon Aβ treatment, but in global terms the number of phosphorylated proteins increased marginally across all the GO categories. This is consistent with Aβ promoting protein phosphorylation and inhibiting protein dephosphorylation.

### Deciphering the Aβ induced phosphointeractome

**Figure 3:**
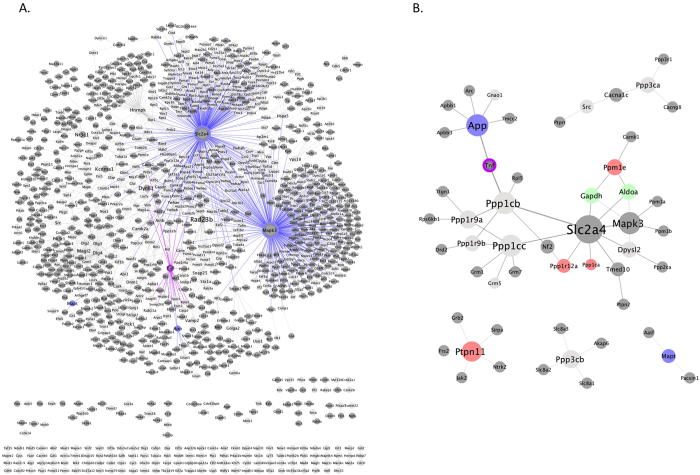
Aβ phosphointeractome. The Aβ responsive phosphointeractome is depicted in (**A**). To obtain the phosphointeractome the phosphorylated and significantly different proteins, recovered experimentally upon Aβ addition, were submitted to IntAct and the interactome produced using Cytoscape 3.3.0. Cluster analysis was carried out using the Cytoscape plugin clusterMaker (GLay). The light grey nodes correspond to the phosphorylated proteins recovered in the experimental procedures and the dark grey nodes their interactors, as identified in IntAct. *Mapt* (encodes the TAU protein) and *App* are represented as blue nodes and the corresponding edges are colour coded also in blue. Two major clusters are identified; with the central nodes *Slc2a4* and *Mapk3* with the circumference and edges in blue. *App* connects to the *Slc2a4* cluster via the *Tnf* node (circumference in purple). A phosphatase sub network is represented in (**B**). (**B**) was extracted from (**A**) by selecting phosphatases, *App, Mapt* and their direct interactors. Bright red nodes were detected only upon Aβ addition (not under basal conditions) and light green nodes represent proteins whose phosphorylation levels decreased significantly upon Aβ addition.

The GpAβ gene list was submitted to IntAct, the interacting proteins identified and the phosphointeractome analyzed using Cytoscape 3.3.0 (Fig. 3A). Data output from IntAct identifies the gene (represented in italics in the text), eventhough the experimental procedures yielding the phosphoproteins and the mass spectrometry data are identifying proteins (normal font in the text). In the resulting Aβ induced phosphointeractome (Fig. 3A) the light grey nodes denote the genes corresponding to the recovered phosphorylated proteins and the dark grey nodes their interactors; identified in IntAct. Of note, phosphorylated TAU (*Mapt*) and phosphorylated APP were recovered (Fig. 3A). In the phosphointeractome two major clusters are readily evident and have as central nodes *Slc2a4* and *Mapk3. App* connects to *Slc2a4* via the *Tnf* node. The former is involved in glucose transport and the latter encodes a serine/threonine kinase.

### Aβ modulates a phosphatase sub network

Given the Aβ phosphatase inhibitory role and the importance of phosphatases in AD^15^,^24^,^26^ pathology, a sub network with respect to protein phosphatases, TAU and APP was elaborated. From the interactome in Fig. 3A, the nodes for phosphatases, *App*, *Mapt* and their direct interactors were extracted, and the sub-network was plotted using cytoscape (Fig. 3B). It is noteworthy that two central nodes, *Slc2a4* and *Mapk3* (dark grey nodes, Fig. 3B) are sustained in both networks (Fig. 3A,B) and bind directly to proteins whose levels of phosphorylation alter significantly following Aβ exposure. In particular, the recovery of phosphorylated GAPDH and ALDOA proteins, decreased upon Aβ addition.

In contrast several phosphorylated proteins appeared ‘de novo’ upon Aβ addition. Two experimentally recovered, protein phosphatases, PPM1E and PTPN11 (Fig. 3B), were found in the GpAβ but not in the GpC (bright red nodes). PTPN11 phosphorylation increased significantly upon Aβ addition and in turn it interacts with another five phosphorylated proteins recovered in the experimental procedures employed.

Furthermore serine/threonine protein phosphatases, and their regulators, appear to be hub nodes in the network depicted in Fig. 3B. *Ppp1ca, Ppp1cb and Ppp1cc* code for serine/threonine-protein phosphatase 1 (PP1) catalytic subunits α, β and γ, respectively. Significantly, PP1α (*Ppp1ca*) is recovered only in GpAβ but not in GpC. That is, phosphorylated peptides for PP1α were identified only when Aβ was added to the primary neuronal cultures (Fig. 3B and Table 1). Peptides for this protein were absent in the mass spectrometry analysis of the GpC, suggesting that phosphorylated PP1α is preferentially found upon Aβ exposure. Activity of these phosphatases is regulated by regulatory subunits of which three were recovered; PPP1R9A, PPP1R9B and PPP1R12A. As mentioned, the latter is a PP1 regulatory subunit recovered in GpAβ but not in GpC.

*Ppp3ca and Ppp3cb* are serine/threonine-protein phosphatase 3 catalytic subunits α and β (PP2B). PP2B is a phosphatase abundantly expressed in the CNS and implicated in AD pathology. It is interesting to note that both of these catalytic subunits were recovered in GpAβ and in GpC (Fig. 3).

Of the above mentioned serine/threonine protein phosphatases, and their regulators, only protein phosphatase PPP1CA and the phosphatase regulatory subunit PPP1R12A increased significantly, precisely by being recovered in their phosphorylated species in response to Aβ exposure. The latter are direct interactors of *Slc2a4*.

### Analysis of Aβ induced phosphointeractome

Using the SysBioTK, phosphoproteins recovered in GpAβ were compared with those recovered in the GpC. One hundred forty one phosphoproteins that significantly change across experimental sets were identified. Phosphorylated proteins whose recovery rate was significantly ‘higher’ (increased) or ‘lower’ (decreased) were identified, herein designated as ‘higher’ or ‘lower’ phosphoproteins. To summarize 73 phosphoproteins were ‘higher’ and 68 ‘lower’, upon Aβ addition (Table 1). Within the ‘lower’ phosphoproteins, 19 were absent in the GpAβ (Aβ ‘lost’), and this was significant when compared to the GpC. In contrast 50 phosphoproteins were recovered only in conditions where Aβ was added (Aβ ‘exclusive’), again these were significantly different across experimental sets, applying the Welch’s t-test (Fig. 1).

**Figure 4:**
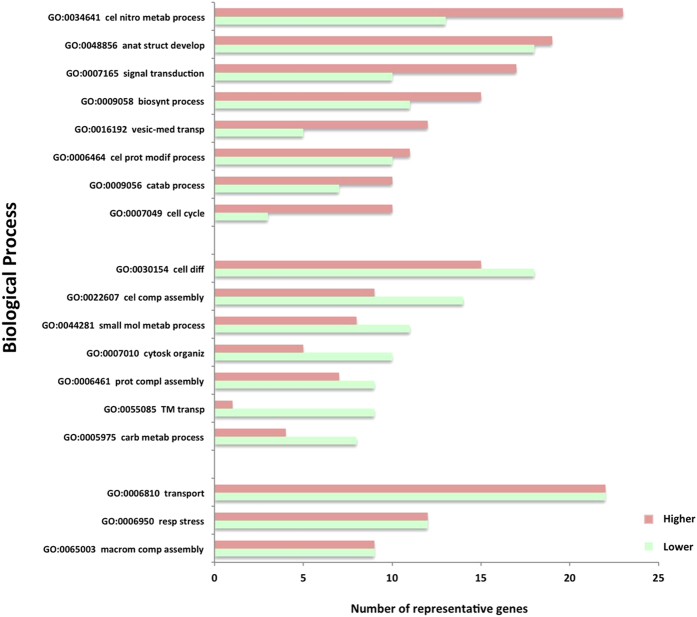
Gene Ontology of significantly different phosphorylated proteins. The two sets of proteins (GpC and GpAβ) were grouped with respect to their Gene Ontology using the SysBioTK for Biological Process. Red bars represent the phosphorylated proteins whose recovery increased upon Aβ addition ‘higher’ phosphoproteins). Green bars represent the phosphorylated proteins whose recovery decreased upon Aβ addition (‘lower’ phosphoproteins). Abbreviations of GOs not in full: GO:0034641 cellular nitrogen compound metabolic process; GO:0048856 anatomical structure development; GO:0007165 signal transduction; GO:0009058 biosynthetic process; GO:0016192 vesicle-mediated transport; GO:0006464 vesicle-mediated transport; GO:0006464 cellular protein modification process; GO:0009056 catabolic process; GO:0030154 cell differentiation; GO:0022607 cellular component assembly; GO:0044281 small molecule metabolic process; GO:0007010 cytoskeleton organization; GO:0006461 protein complex assembly; GO:0055085 transmembrane transport; GO:0005975 carbohydrate metabolic process; GO:0006950 response to stress; GO:0065003 macromolecular complex assembly.

The GO of significantly different ‘higher’ and ‘lower’ phosphoproteins were analyzed for Biological Process (Fig. 4). The top groups, for Biological Processes, are those where the ‘higher’ phosphoproteins are greater than the ‘lower’ phosphoproteins, the net effect is an increase in phosphorylated proteins. Top functions are consistent with functions described for APP and furthermore, have been associated with AD, for example signal transduction and vesicle-mediated transport.

The middle group of Biological Processes, are those where the ‘lower’ phosphoproteins are greater than the ‘higher’ phosphoproteins, the net effect is a decrease in phosphorylated proteins (Fig. 4). Top functions within this group, include small molecule metabolic processes, cytoskeletal organization and transmembrane transport, these processes have also been associated with AD.

In the bottom group (Fig. 4), the number of ‘higher’ and ‘lower’ phosphoproteins is similar. However although the number of proteins is sustained, the proteins are different. To better understand the underlying molecular processes, proteins were analyzed as described below.

### Interactome of significantly different phosphoproteins upon Aβ exposure

**Figure 5:**
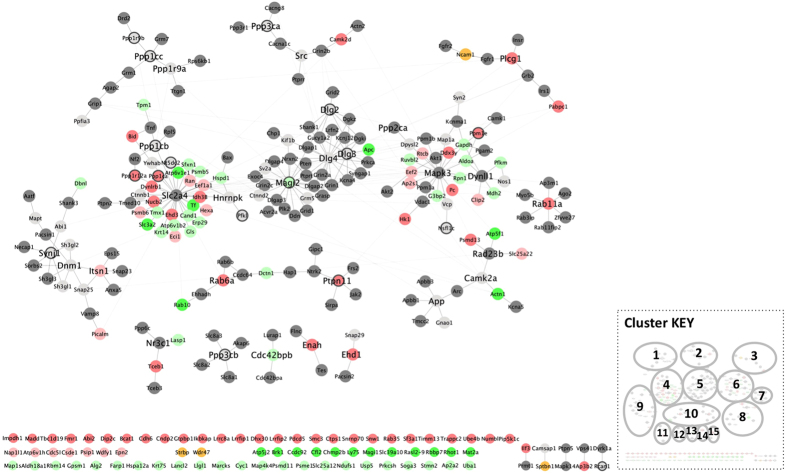
Aβ induced phospho network. Figure 5 represents a subset from Fig. 3, retaining the nodes of significantly different phosphoproteins, as well as those with the term ‘protein phosphatase’ in their GO (black contour). Directly interacting nodes of the latter were maintained. The interacting proteins, that were identified in the experimental set up are coloured light grey; other interacting proteins are dark grey. Dark yellow nodes represent proteins that both increased and decreased upon Aβ addition (see Supplementary Table S3). ‘Higher’ phosphoproteins are light red and phosphoproteins recovered only in conditions where Aβ was added (Aβ ‘exclusive’) are bright red. ‘Lower’ phosphoproteins are light green and phosphoproteins not recovered upon Aβ addition (Aβ ‘lost’) are bright green. The network was produced using Cytoscape 3.3.0 and cluster analysis was carried out using the Cytoscape plugin clusterMaker (GLay).

From the Aβ phosphointeractome (Fig. 3A), a simplified network was developed using subsets of nodes (Fig. 5). The following classes of nodes and their direct interactors were selected; genes corresponding to significantly ‘higher’ and ‘lower’ phosphoproteins identified across experimental sets (listed in Table 1), as well as those from the GpAβ with the GO term ‘protein phosphatase’. The nodes *App* and *Mapt* were also included. The interactions were plotted as a network using Cytoscape 3.3.0 (Fig. 5 and Supplementary Table S2). Bright green nodes correspond to Aβ ‘lost’ phosphoproteins and bright red nodes to Aβ ‘exclusive’ phosphoproteins comparative to Control conditions. Fifteen clusters were identified with the Cytoscape plugin clusterMaker (GLay).

### Unaltered phosphoproteins as central nodes

Clusters 1 (*Ppp1cc*), 2 (*Ppp3ca*) and 12 (*Ppp3cb*) include phosphatases (Fig. 5 and Supplementary Table S2) that have already been discussed and whose phosphorylation levels do not alter significantly upon Aβ addition. An exception is the *Ppp3ca* cluster (cluster 2) where CAMK2D exhibits ‘higher’ phosphorylation. Cluster 12 includes nodes *Ppp3cb* and *Slc8a3*, evoking putative functional links to long-term memory potentiation, given the biological function of these proteins. These functions are significantly compromised in AD, and it is relevant that the Aβ induced phospho network includes these nodes (Fig. 5).

Clusters 9 (*Synj1*) and 8 (*Camk2a*) include *Mapt* and *App* respectively (Fig. 5 and Supplementary Table S2). Other nodes in cluster 9, include *Dnm1, Itsn1, Picalm and Dbnl*. ITSN1 and PICALM, revealed ‘higher’ phosphorylation levels upon Aβ addition, in contrast DBNL has ‘lower’ levels.

The central node in cluster 8 is *Camk2a*, crucial for plasticity at glutamatergic synapses. This calcium calmodulin-dependent protein kinase is composed of four different chains: alpha, beta, gamma, and delta. CAMK2A interacts directly with ACTN1, but following Aβ addition the phosphorylated form of the latter is no longer recovered. ACTN1 is involved in the regulation of the actin cytoskeleton. *Atp5f1* is another Aβ ‘lost’ phosphoprotein in this cluster, in contrast proteins encoded by *Slc25a22* and *Psmd13* show ‘higher’ phosphorylated levels in response to Aβ, the latter is Aβ ‘exclusive’.

Cluster 5 (*Dlg2/Dlg3/Dlg4*) includes DLG family members (Fig. 5 and Supplementary Table S2), which are essential for maintaining synaptic architecture and plasticity. These proteins although well represented as phosphoproteins in the primary neuronal cell cultures, did not alter significantly upon Aβ exposure (dark grey nodes).

### ‘Lower’ and ‘Higher’ phosphoproteins as central nodes

The *Dlg* cluster 5 has another central node, *Magi2* (Fig. 5 and Supplementary Table S2). MAGI2 is a ‘lower’ phosphoprotein also involved in development and maintenance of the synapse. Even though cluster 5 does not have a substantial number of nodes that are significantly altered following Aβ exposure, the phosphorylated protein APC is Aβ ‘lost’. *Cdc42bpb* is another central node (cluster 13) exhibiting ‘lower’ phosphorylation across experimental sets.

Clusters with central nodes that represent ‘higher’ phosphoproteins, are in general terms smaller (Fig. 5 and Supplementary Table S2); these include clusters 14 (*Enah*), 15 (*Ehd1*), 3 (*Plcg1*), 7 (*Rab11a*) and 10 (*Rab6a*). *Plcg1* is involved in regulating intracellular signalling cascades and in this cluster besides *Pabpc*1, *Ncam 1* also exhibited ‘higher’ recovery of the phosphorylated proteins. Two clusters have as central nodes RAB11a (cluster 7) and RAB6a (cluster 10), these proteins are small GTPases involved in intracellular membrane trafficking. *Rab11a* is Aβ ‘exclusive’ *and Rab6a* represents likewise an Aβ ‘exclusive’ ‘higher’ phosphoprotein. Another ‘higher’ phosphoprotein identified in the latter cluster is PTPN11, already discussed. In contrast RAB10 (cluster 10) is Aβ ‘lost’ and phosphorylated DCTN1 is significantly ‘lower’.

### Bioinformatically identified central nodes

Three clusters 11, 4 and 6, have central nodes, which were not experimentally identified but became evident following the bioinformatics analysis. Cluster 11 (*Nr3c1*) is a small cluster and includes PPP6C, LASP1 a ‘lower’ phosphoprotein and TCEB1 that is Aβ ‘exclusive’. Clusters 4 (*Slc2a4*) and 6 (*MapK3*), with central nodes already discussed, contain the greatest number of significantly different phosphoproteins. Taken together they include three Aβ ‘lost’ phosphoproteins and eleven Aβ ‘exclusive’.

### Significantly different phosphoprotein network

**Figure 6:**
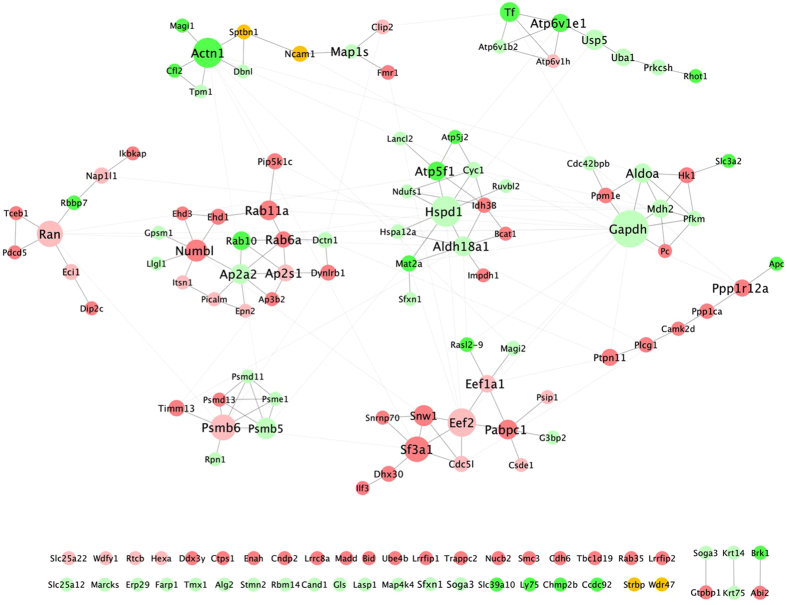
Aβ induced significantly different phospho network. Protein interactions between phosphorylated proteins, whose recovery significantly increased (‘higher’) or decreased (‘lower’) following Aβ addition, were mapped using STRING. ‘Higher’ phosphoproteins are light red and phosphoproteins recovered only in conditions where Aβ was added (Aβ ‘exclusive’) are bright red. ‘Lower’ phosphoproteins are light green and phosphoproteins not recovered upon Aβ addition (Aβ ‘lost’) are bright green. Dark yellow nodes represent the same protein, but distinct identifiers that increased and decreased in response to Aβ addition (Supplementary Table S3). The network was produced using Cytoscape 3.3.0.

Many of the ‘higher’ and ‘lower’ phosphoproteins have been reported to interact, thus the identifiers in Table 1 were submitted to STRING and further interactions identified (Fig. 6). Nine clusters are particularly evident, with the central nodes *Actn1, Atp6v1e1, Gapdh, Hspd1, Rab11a/Numbl, Ran, Ppp1r12a, Eef2/Sfea1* and *Psmb6*. The first four clusters include predominantly ‘lower’ phosphoproteins and the last five, predominantly ‘higher’ phosphoproteins. Clusters were organized by degree such that the central nodes have the greatest number of edges and thus are the most likely key genes with respect to Aβ induced responses. Hence the proteins they encode represent strong AD biomarker candidates, given that the recovery of the phosphorylated protein across experimental sets was significantly decreased or increased upon Aβ addition. In some cases the recovery of the phosphoprotein was completely ‘lost’ or ‘exclusive’ following conditions of Aβ addition (bright green and bright red nodes respectively, Fig. 6).

Many of the above mentioned central nodes have already been discussed, but close analysis of Fig. 6 reveals key cellular processes following Aβ exposure. For example Aβ induced reduced phosphorylation levels of; the *Actn1* cluster which implies targeting cytoskeletal organization; the *Atp6v1e1* cluster impacting lysosomal, iron transport and ubiquitination processes; and the *Hspd1* cluster which is associated with the heat shock response.

Another cluster includes *Gapdh*, which plays a role in glycolysis and nuclear functions. This cluster has three phosphorylated proteins which are significantly ‘higher’ and five which are significantly ‘lower’ (Fig. 6), among them *Aldoa;* a glycolytic enzyme.

As previously mentioned Aβ causes ‘higher’ *Rab6a* and *Rab11a* phosphorylation, but *Rab10* is Aβ ‘lost’. Another phosphoprotein significantly decreased is AP2A2, a component of the adaptor protein complex 2 (AP2) involved in endocytosis related processes.

The *Ppp1r12a* cluster is particularly interesting from a biomarker standpoint as it includes 5 ‘higher’ Aβ ‘exclusive’ phosphoproteins. This is also the case for the *eEf2* cluster. The latter is a member of the GTP-binding translation elongation factor family and an essential factor for protein synthesis.

## Discussion

Identification of significantly ‘higher’ and ‘lower’ phosphoproteins in response to Aβ exposure, proved to be an important approach to providing key biomarker candidates. Analysis of the proteins, identified across experimental sets, with respect to Biological Process identified crucial processes that were corroborated, when the functions of the phosphorylated proteins themselves were investigated. Among the most recurring processes are signal transduction, endocytosis, cytoskeletal organization and intracellular transport. Alterations in these cellular events have in turn, all been associated with AD.

The phosphorylation levels of many proteins involved in membrane trafficking changed, as is the case of ITSN1 whose phosphorylation levels increases in response to Aβ. ITSN1 has been implicated in Down’s syndrome and AD, possibly via c-JUN N terminal kinase activation^27^. It is a cytoplasmic membrane-associated protein involved in endocytic membrane traffic and appears to regulate the formation of clathrin-coated vesicles and to be involved in synaptic vesicle recycling. Furthermore, ITSN1 was shown to interact with AP2^28^. Interestingly, AP2A2, a component of the AP2 adaptor complex, is a phosphoprotein significantly decreased upon Aβ exposure. This complex is also involved in clathrin-dependent endocytosis and serves as a cargo receptor, selectively sorting the membrane proteins involved in receptor-mediated endocytosis. The *AP2A2* gene has been associated with AD^29^. Furthermore it is noteworthy that the complex AP2/*PICALM*, interacts with APP directing it to degradation and autophagy^30^. Given the important roles of endocytosis and Aβ production in AD, genes impacting this process are extremely relevant.

RABs comprise a subfamily of small GTPases also involved in the regulation of several steps during membrane trafficking, including vesicle formation, movement along the cytoskeleton network and fusion at the target membrane. *Rab10* codes for an Aβ ‘lost’ phosphoprotein while *Rab11a*, cluster 7 in Fig. 5, is Aβ ‘exclusive’ and is significantly associated with late-onset AD^31^. *Rab6a* is likewise an Aβ ‘exclusive’ ‘higher’ phosphoprotein. RAB6 was shown to regulate intracellular APP processing and trafficking. Furthermore, upregulation of RAB6A in AD is linked to ER stress^32^. Aβ can affect the phosphorylation states of many proteins involved in diverse cellular processes and induces stress. As an example, another central cluster includes *Gapdh and Aldoa.* GAPDH interacts with APP, and there is significant inhibition of the former in AD^33^. Further, oxidative modification appears to be a relevant neurotoxic pathway in AD cases correlated with GADPH. Alterations of ALDOA have also been associated with AD^34^.

Since APP processing and Aβ production involve intracellular transport and vesicle-mediated transport^35^,^36^, one can hypothesize that Aβ may be involved in regulating its own production, via modulating phosphorylation of proteins involved in the above mentioned cellular processes. The peptide was reported to alter APP nuclear signalling^37^ and to impair APP secretion/vesicular anterograde transport and exocytosis, through a mechanism mediated by altered cytoskeleton dynamics of both microtubule and actin networks^25^,^38^.

Indeed, the actin cytoskeleton is also relevant for synaptic remodeling and AD pathogenesis^25^. Two cytoskeletal related phosphoproteins whose recovery rate decreased were identified. ACTN1 is a bundling protein and an F-actin cross-linking protein thought to anchor actin to a variety of intracellular structures. It is involved in the regulation of the actin cytoskeleton and is an Aβ “exclusive” phosphoprotein. Reports have associated *ACTN1* to AD^39^. *Cdc42bpb* is a central node (cluster 13) exhibiting ‘lower’ phosphorylation. It regulates actin cytoskeletal organization and is a downstream effector of CDC42, whose activity is increased upon Aβ treatment^40^.

Aβ affected the phosphorylation level of proteins directly involved in synaptic signalling. In particular, MAGI2 is a ‘lower’ phosphoprotein. It is a scaffold molecule at synaptic junctions and assembles neurotransmitter receptors and cell adhesion proteins. It appears to be essential for development and maintenance of the synapse, binding to other scaffold proteins supporting cell junctions^41^. *MAGI2* and AD have not being extensively explored but genome-wide association studies placed this gene as a candidate locus in the etiology of sporadic AD.

Further, several members of the DLG family were also found, even though their phosphorylation did not change significantly in response to Aβ. These proteins can be recruited to the post-synaptic density, where they are essential for maintaining synaptic architecture and plasticity. Both *DLG3* (encodes SAP-102) and *DLG4* (encodes PSD-95) have been associated with AD; their protein levels are reduced in AD brains^42^. Further, given that *DLG3* and *DLG4* encode post-synaptic scaffold proteins, which regulate NMDA receptor synaptic activity and expression, this presents a possible mechanism for aberrant expression in AD. NMDA receptor-evoked excitotoxicity contributes to glutamatergic synapses mediating cognitive decline in AD^42^. *SYNJ1* is another protein whose phosphorylation levels did not change but its expression is likely to affect synaptic transmission and membrane trafficking. These are also hallmarks of the disease and SYNJ1 was reported to increase in AD^43^.

Although not found under our experimental conditions, two central genes linking many altered phosphoproteins were identified by the IntAct searches; the Slc2a4 and *MapK3* nodes*. Slc2a4* encodes a protein that functions as an insulin-regulated facilitative glucose transporter. Altered metabolism of brain glucose has been suggested in diabetes and AD^44^. Of note, decreased SLC2A4 expression has been observed in adipose tissue from type 2 diabetic patients and diabetes is increasingly associated with AD. *MapK3* encodes a serine/threonine kinase; an essential component of the Map kinase signal transduction pathway. The MAPK/ERK cascade regulates many biological functions, such as cell adhesion, survival, growth and differentiation, regulation of transcription and translation, and cytoskeletal rearrangements. Moreover, this cascade regulates endosomal dynamics, lysosome processing and endosome cycling; these processes, have been associated with APP, TAU and AD. Increased activities and anomalies on MAPK signalling have been closely associated to disease pathology^45^.

The Aβ impact on both kinases and phosphatases is well described. In this work a network of phosphatases whose phosphorylation increases in response to Aβ was recovered. *Ptpn11* (protein tyrosine phosphatase, non-receptor type 11) is a “higher” phosphoprotein that interacts with many genes/proteins likewise altered. Ptpn11 encodes a signalling molecule involved in activation of the RAS/MAPK pathway and STAT signalling pathways. *PTPN11* has been tagged as a hub gene in AD^46^. One can therefore deduce that phosphorylated PTPN11 should be investigated as a potential AD biomarker. Of note, *Plcg*1, an Aβ “exclusive” protein, can become activated in response to ligand-mediated activation of receptor-type tyrosine kinases and is involved in regulating intracellular signalling cascades. Further, *PLCG1* has been tagged as a hub gene in AD^46^.

Noticeably, PP1α (*Ppp1ca*) is another phosphatase recovered only in GpAβ. PP1 is an abundant neuronal phosphatase enriched in dendritic spines^47^,^48^ with a key role in synaptic signalling; and it can be inhibited by Aβ. PP1 is required for long-term depression, is involved in memory and learning and has been implicated in TAU dephosphorylation, playing a key role in AD pathogenesis. Interestingly, the activity of phosphatases can be regulated by different regulatory subunits. PPP1R12A is a PP1 regulatory subunit recovered in GpAβ but not in GpC.

*Ppm1e* encodes a member of the PP2C family of the serine/threonine-protein phosphatases that also exhibits increased phosphorylation. It is brain-specific, involved in synaptic plasticity and dendritic spine morphogenesis and negatively regulates the Ca^2+^/calmodulin dependent kinases (CaMK) IV and II and the p21-activated kinase (PAK) 1; kinases important in actin cytoskeletal regulation. This phosphoprotein may likewise represent an interesting target in AD pathology.

In conclusion, due to the dynamic nature of protein phosphorylation systems, where the phosphorylation of a given protein can evoke the phosphorylation of further proteins, it is not surprising that a given cluster can have both ‘higher’ and ‘lower’ phosphoproteins. This work clearly showed that Aβ altered the phosphorylation levels of many proteins and this is consistent with the pathophysiological characteristics attributed to Aβ, placing it at the center of AD. From the dataset here presented it was possible to identify 141 putative biomarkers, whose phosphorylated proteins significantly increased (73) or decreased (68) upon Aβ addition across experimental sets. Furthermore 19 phosphoproteins were ‘lost’ upon Aβ exposure and 50 were ‘exclusive’ to Aβ addition. These proteins and their levels of phosphorylation provide a resource as potential phospho biomarker candidates for AD diagnosis and should be pursued in this respect in future studies.

## Online Methods

### Neuronal primary cultures

Primary cortical neuronal cultures were prepared from Wistar Hannover rat embryo at 18^th^ day of gestation as previously described^37^. Briefly, cerebral cortex was dissected and dissociated with trypsin (0,23 mg/mL) and desoxyribonuclease I (0,15 mg/mL) in Hanks balanced solution (HBSS). Cells were then plated onto poly-D-lysine coated dishes at 6 × 10^6^ cells/100 mm density in Neurobasal medium (Gibco) supplemented with a serum-free medium combination of B27 (NB-B27), glutamine (0,5 mM) and gentamicin (60 μg/mL). Cells were maintained in an atmosphere of 5% CO_2_ at 37 °C and experiments were carried out on the 10^th^ day of primary neuronal cultures *in vitro*.

All experimental procedures followed the European legislation for animal experimentation (2010/63/EU) and no specific ethics approval under EU guidelines was required. This is within the European law (Council Directive 86/609/EEC) and during the procedure all steps were taken to ameliorate animal suffering. Procedures were approved and supervised by the Institutional Animal Care and Use Committee: Comissão Responsável pela Experimentação e Bem-Estar Animal (CREBEA).

### Aβ treatment and phosphorylation mimicking conditions

Aβ1-42 peptide (American peptide) was aggregated in PBS for 48 h at 37 °C (100 μM aggregated stock) as previously described^49^. After aggregation, Aβ was added to primary neuronal cultures (Fig. 1), as a 10 μM solution in NB-B27 medium, for 3 h. Cell lysates were collected and the samples were processed for phosphoprotein enrichment as described below.

### Phosphoprotein enrichment

Phosphoprotein enrichment was performed using phosphate metal affinity chromatography (TALON^®^ PMAC Phosphoprotein Enrichment Kit, Clontech) columns, which allows for the selective binding of proteins that contain a phosphate group on any amino acid (including serine, threonine or tyrosine), according to the manufacturer’s instructions. Briefly, after the specified treatments, cells were washed with PBS, scrapped, centrifuged at 500 g for 5 min and the resulting pellet was frozen at −80 °C. Extraction/loading buffer was added to each sample according to pellet weight (30 μL buffer A/1mg of pellet), supplemented with sodium fluoride (a phosphatase inhibitor) to a final concentration of 10 mM. The samples were then incubated at 4 °C for 10 min and centrifuged at 10,000 g, for 20 min at 4 °C. In parallel, columns were washed with distilled water and twice with extraction/loading buffer to equilibrate the columns.

The supernatants obtained by centrifugation (total phosphoproteins extracts) were added to the columns and shaken at 4 °C for 20 min, for phosphoprotein binding. After 4 washes, phosphorylated proteins were eluted using 1 ml of buffer B, for 4 times. From the 4 protein fractions obtained, fraction 2 contained the most enriched fraction, as determined by BCA assay (pierce). Samples were stored at −80 °C until lyophilization for MS analysis (Fig. 1).

### MS/MS Analysis and protein identification

Lyophilized samples were dissolved in lithium dodecyl sulfate (LDS) buffer, incubated at 95 °C for 10 min, sonicated and loaded onto a one-dimensional polyacrylamide gel (1DE) system. Trypsin digestion of proteins to peptides occurred after 1DE staining with Coomassie blue and excision of the protein bands. Peptides were extracted from the gels using trifluoroacetic acid (TFA) 0,1% + ACN (50:50), and the resulting supernatant dried in SpeedVac, ressuspended in TFA 0.1% and stored at −20 °C. Proteolytic samples were injected in the Q Exactive-Orbitrap LC-MS/MS System (Thermo Scientific) and MS/MS spectra data acquisition followed by phosphoprotein identification^50^,^51^,^52^, using proteome discovery software (Thermo Scientific). Semi-quantitative analysis of the data was carried out using the International Protein Index (IPI) database for protein search and Rattus novergicus as the organism model. A false discovery rate of 1% was applied. A single phosphopeptide identification was set to be sufficient for phosphoprotein identification. Further data analysis was done similar to the black-and-white method used before^50^,^52^. Phosphoproteins resulting from each of the experiments were analyzed as described below.

### Bioinformatic and Statistical Analysis for phosphoprotein characterization using the SysBioTK library

The Systems Biology Toolkit (SysBioTK) library was employed to analyze the data from each of the experimental conditions. For details see da Cruz e Silva, 2016 (submitted), available at https://bitbucket.org/CrisXed/sysbiotk. For the statistical analysis, each iteration of the experiment is considered a dataset. As a first step, the yield in terms of number of proteins for each dataset was used to identify the outlier datasets, using the modified Thompson tau, τ, test with a confidence level of 95%. Subsequently, the protein IPI accession numbers were converted to the corresponding UniProt accession numbers. For this process, the cross references from the IPI database were used to identify the UniProt/Swiss-Prot accession numbers corresponding to each IPI accession number. For IPI accession numbers not identified, the SysBioTK BLAST+ parser was employed, with a similarity parameter of 0.90. The utility performs a blast search against a subset of the UniProt database (Swiss-Prot accession numbers, organism rattus), effectively identifying UniProt/SwissProt accession numbers corresponding to the IPI accession numbers. The process was then repeated, starting with the cross references, but against the UniProt/TrEMBL databases and only for the IPI accession numbers without a corresponding UniProt/SwissProt accession number. The analysis was carried out on the 27^th^ of September 2015.

The datasets were grouped into two groups, one for each experimental condition (control and exposure to Aβ). The SysBioTK statistical analysis was employed to identify, between the two groups, which accession numbers were significantly different. For this purpose, the tool employed Welch’s t-Test with a confidence level of 95%. Consequently, two protein lists were obtained; those where the retrieval of the phosphoprotein showed a significant decrease upon addition of Aβ (‘lower’ phosphoproteins), and those where the retrieval of the phosphoprotein showed a significant increase upon addition of Aβ (‘higher’ phosphoproteins). To produce the interacting networks, accession number lists were submitted to IntAct on the 29^th^ of October 2015 and the information was loaded into Cytoscape 3.3.0^53^. Cluster analysis was carried out using the Cytoscape plugin clusterMaker (GLay). Significantly different phosphoproteins were also submitted to STRING, on the 3^rd^ of December 2015, and resulting interactions were plotted using Cytoscape 3.3.0.

## Additional Information

**How to cite this article**: Henriques, A. G. *et al*. Altered protein phosphorylation as a resource for potential AD biomarkers. *Sci. Rep.* **6**, 30319; doi: 10.1038/srep30319 (2016).

## Supplementary Material

Supplementary Information

## Figures and Tables

**Table 1:** Significantly different recovery rates of phosphoproteins upon Aβ addition.

*‘Lower’ Phosphoproteins*			*‘Higher’ Phosphoproteins*		
***Actn1***	Hspa12a	Rbm14	**Abi2**	**Gtpbp1**	Psip1
Aldh18a1	Hspd1	***Rhot1***	Ap2s1	Hexa	Psmb6
Aldoa	Krt14	Rpn1	**Ap3b2**	**Hk1**	**Psmd13**
Alg2	Krt75	Ruvbl2	Atp6v1h	**Idh3B**	**Ptpn11**
Ap2a2	Lancl2	Sfxn1	**Bcat1**	**Ikbkap**	**Rab11a**
***Apc***	Lasp1	Slc25a12	**Bid**	**Ilf3**	**Rab35**
***Atp5f1***	Llgl1	***Slc39a10***	**Camk2d**	**Impdh1**	**Rab6a**
***Atp5j2***	***Ly75***	***Slc3a2***	Cdc5l	Itsn1	Ran
Atp6v1b2	***Magi1***	Soga3	**Cdh6**	**Lrrc8a**	Rtcb
***Atp6v1e1***	Magi2	Sptbn1	Clip2	**Lrrfip1**	**Sf3a1**
***Brk1***	Map1s	Stmn2	**Cndp2**	**Lrrfip2**	Slc25a22
Cand1	Map4k4	Strbp	Csde1	**Madd**	**Smc3**
***Ccdc92***	Marcks	***Tf***	**Ctps1**	Nap1l1	**Snrnp70**
Cdc42bpb	***Mat2a***	Tmx1	**Ddx3y**	*Ncam1*	**Snw1**
***Cfl2***	Mdh2	Tpm1	**Dhx30**	**Nucb2**	*Sptbn1*
***Chmp2b***	Ncam1	Uba1	**Dip2c**	**Numbl**	*Strbp*
Cyc1	Ndufs1	Usp5	**Dynlrb1**	**Pabpc1**	**Tbc1d19**
Dbnl	Pfkm	Wdr47	Eci1	**Pc**	**Tceb1**
Dctn1	Prkcsh		Eef1a1	**Pdcd5**	**Timm13**
Erp29	Psmb5		Eef2	Picalm	**Trappc2**
Farp1	Psmd11		**Ehd1**	**Pip5k1c**	**Ube4b**
G3bp2	Psme1		**Ehd3**	**Plcg1**	Wdfy1
Gapdh	***Rab10***		**Enah**	**Ppm1e**	*Wdr47*
Gls	***Rasl2-9***		Epn2	**Ppp1ca**	
Gpsm1	***Rbbp7***		**Fmr1**	**Ppp1r12a**	

Upon Aβ addition the number of phosphorylated proteins recovered decreased significantly (‘lower’ Phosphoproteins) while others increased (‘higher’ Phosphoproteins). The genes encoding these significantly different proteins were identified using the SysBioTK (Welch’s t-test). Genes underlined correspond to the same protein recovered under both conditions, although different identifiers were involved (see Supplementary Table S2). Genes in bold and italics correspond to phosphoproteins present under control conditions but absent (Aβ ‘lost’) upon Aβ addition and genes in bold correspond to phosphoproteins detected only upon addition of Aβ (Aβ ‘exclusive’).
