# Supplementary material for: Altered protein phosphorylation as a resource for potential AD biomarkers
Source: Sci Rep. 2016 Jul 28;6:30319. doi: 10.1038/srep30319 (PMC4964585; doi:10.1038/srep30319)
Supplement: Supplementary Information [file srep30319-s1.pdf]

Supplementary material

Article Tittle:
Altered protein phosphorylation as a resource for potential AD biomarkers

Ana Gabriela Henriques[1*#], Thorsten Muller[2#], Joana Machado Oliveira[1], Marta Cova[1], Cristóvão B. da Cruz e Silva[3] and Odete A. B. da Cruz e Silva[1]

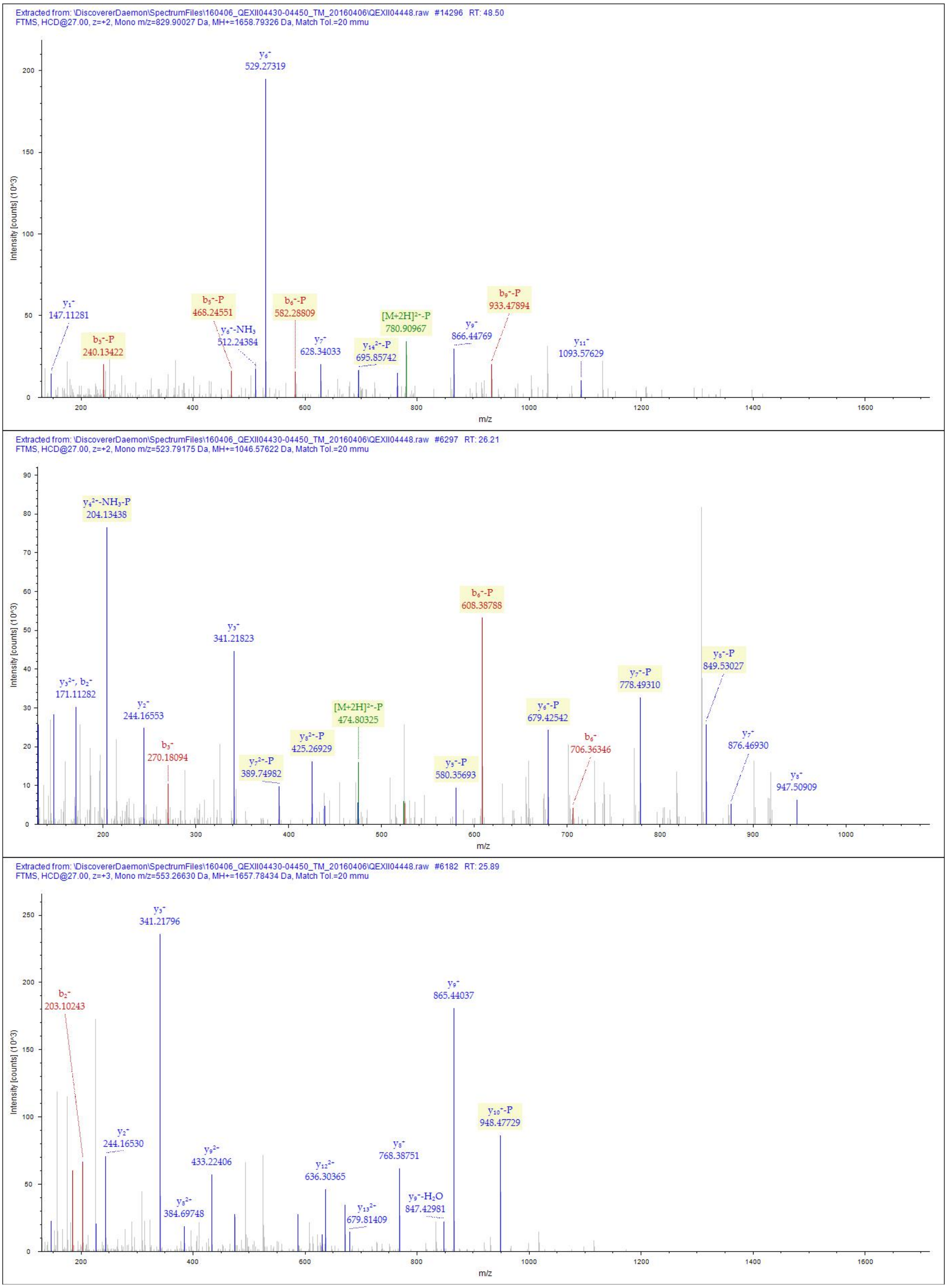

Supplementary Fig. S1. Example of 3 identified phosphopeptides for Mapt.
MSMS spectra for peptides IGsLDNITHVPGGGNK, VAVVRtPPK and TTPSPKtPPGSGEPPK are demonstrated.

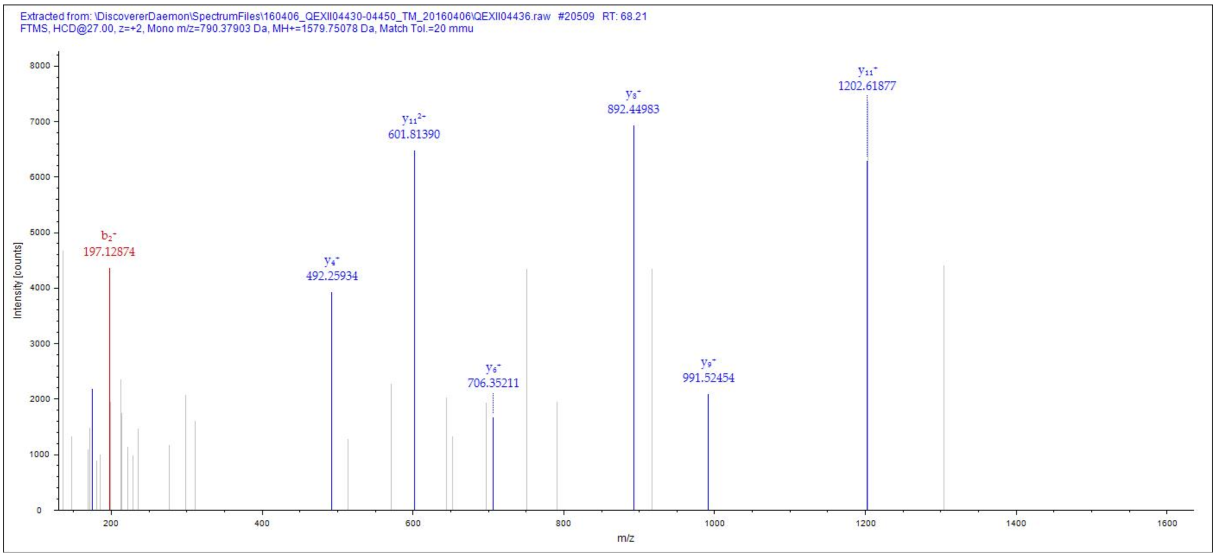

Supplementary Fig. S2. Example of an identified phosphopeptide for Gapdh. A phosphopeptide VPtPNVSVVDLTCR of GAPDH is shown, where the threonine at residue 3 is phosphorylated (precursor mass m/z=790.37903 Da).

Supplementary Table S1. Phosphoproteins recovered from phospho columns. The numbers of phosphoproteins (PP) obtained upon addition of Aβ or under basal conditions (PP Control) are indicated for each experimental set (Exp. #). Significantly different results were identified by applying the modified Thompson tau, τ, test and outliers removed (*). The total number of proteins (excluding duplicates) carried forward to subsequent analysis is indicated.

Exp. #	PP Control	PP Abeta
1	505	543
2	1062*	834
3	554	381
4	455	451
5	569	682
6	513	208
Total	870	986

Supplementary Table S2. Principal clusters in the Aβ-induced phosphoprotein network. Nodes present in the 15 clusters represented in Fig. 5 are detailed. Clusters are numbered and the total and central nodes in each identified. Nodes are subdivided as to whether they were identified as interactors in intAct or in the phospho column. Significantly Higher and 'lower' phosphoproteins, as well as those 'lost' or 'exclusive' to Aβ addition, are also identified. Nodes in bold are the central nodes.

Cluster	**1**<br>11 nodes	**2**<br>9 nodes	**3**<br>8 nodes	**4**<br>38 nodes	**5**<br>36 nodes	**6**<br>32 nodes	**7**<br>7 nodes	**8**<br>13 nodes	**9**<br>20 nodes	**10**<br>13 nodes	**11**<br>5 nodes	**12**<br>5 nodes	**13**<br>3 nodes	**14**<br>3 nodes	**15**<br>3 nodes
**Central nodes**	Ppp1cc<br>Ppp1r9a	Ppp3ca<br>Src	Plcg1	Hnrnpk<br>Ppp1cb<br>Slc2a4	Dlg2<br>Dlg3<br>Dlg4<br>Magi2	Dynll1<br>Mapk3<br>Ppp2ca	Rab11a	App<br>Camk2a<br>Rad23b	Dnm1<br>Itsn1<br>Synj1	Ptpn11<br>Rab6a	Nr3c1	PPP3cb	Cdc42bpb	Enah	Ehd1
**IntAct**	Agap2<br>Rps6kb1<br>Grm1<br>Grm7<br>Drd2<br>Ttgn1<br>Grip1	Actn2<br>Cacna1c<br>Cacng8<br>Grin2b	Fgfr1<br>Fgfr2<br>Grb2<br>Insr<br>Irs1	**Slc2a4**<br>Bax<br>Nf2<br>Rpl5<br>Tmed10<br>Tnf	Acvr2a<br>Chp1<br>Ddn<br>Dgki<br>Dgkz<br>Dlgap1<br>Dlgap2<br>Dlgap3<br>Dlgap4<br>Exoc4<br>Grasp<br>Grid1<br>Grid2<br>Grin1<br>Grin2a<br>Grin2c<br>Gucy1a2<br>Kcna4<br>Kcnj12<br>Lrfn2<br>Nrxn2<br>Plk2<br>Prkca<br>Pten<br>Ptprt<br>Shank1<br>Syngap1	**Dynll1**<br>**Mapk3**<br>**Ppp2ca**<br>Akt1<br>Akt2<br>Camk1<br>Kcnma1<br>Pgam2<br>Vdac1	Rab3ip<br>Ago2<br>Zfyve27<br>Ap3m1<br>Myo5b<br>Rab11fip2	Rad23b<br>Apbb1<br>Apbb3<br>Arc<br>Kcna5<br>Tmcc2	Aatf<br>Anxa5<br>Eps15<br>Necap1<br>Pacsin1<br>Sh3gl3<br>Shank3<br>Snap23<br>Sorbs2<br>Vamp8	Ccdc64<br>Ehhadh<br>Frs2<br>Gipc1<br>Hap1<br>Jak2<br>Ntrk2<br>Rab6b<br>Sirpa	**Nr3c1**<br>Tceb3	Akap6<br>Slc8a1<br>Slc8a2<br>Slc8a3	Cdc42bpa<br>Lurap1	Flnc<br>Tes	Pacsin2
**Phos Column**	**Ppp1cc**<br>**Ppp1r9a**<br>Ppp1r9b<br>Ppfia3	**Ppp3ca**<br>**Src**<br>Ppp3r1<br>Ptprr	*Ncam1	**Hnrnpk**<br>**Ppp1cb**<br>Nt5dc2<br>Ywhab<br>Ctnnb1<br>Ptpn2<br>Pfkl	**Dlg2**<br>**Dlg3**<br>**Dlg4**<br>Ctnnd2<br>Grm5<br>Kif1b<br>Sv2a	Dpysl2<br>Map1a<br>Nos1<br>Nsfl1c<br>Ppm1a<br>Ppm1b<br>Syn2<br>Vcp		**App**<br>**Camk2a**<br>Gnao1	**Dnm1**<br>**Synj1**<br>Abi1<br>Mapt<br>Sh3gl1<br>Sh3gl2<br>Snap25		Ppp6c	**Ppp3cb**			Snap29
**Lower**				Atp6v1b2<br>Cand1<br>Erp29<br>Gls<br>Hspd1<br>Krt14<br>Psmb5<br>Sfxn1<br>Tmx1<br>Tpm1	**Magi2**	Aldoa<br>G3bp2<br>Gapdh<br>Mdh2<br>Pfkm<br>Rpn1<br>Ruvbl2			Dbnl	Dctn1	Lasp1		**Cdc42bpb**		
**Aβ lost**				Atp6v1e1<br>Slc3a2<br>Tf	Apc			Actn1<br>Atp5f1		Rab10					
**Higher**				Eci1<br>Eef1a1<br>Hexa<br>Psmb6<br>Ran		Ap2s1<br>Clip2<br>Eef2<br>Rtcb		Slc25a22	**Itsn1**<br>Picalm						
**Aβ exclusive**		Camk2d	**Plcg1**<br>Pabpc1	Bid<br>Ppp1ca<br>Ppp1r12a<br>Dynlrb1<br>Nucb2<br>Ehd3<br>Idh3B		Ddx3y<br>Hk1<br>Pc<br>Ppm1e	**Rab11a**	Psmd13		**Ptpn11**<br>**Rab6a**	Tceb1			**Enah**	**Ehd1**

Supplementary Table S3. Dual response proteins. Different identifiers (UniProt (IPI)) corresponding to the same gene resulted in both 'higher' and 'lower' levels of phosphorylated protein. The public databases used did not distinguish the IPIs as different proteins. Consequently the corresponding IPI sequences were aligned using BLAST and in all cases differences (in red) were identified, which may explain the dual response for 'higher' and 'lower' phosphorylation recoveries for the same protein.

Gene	Sig Dif	Identifiers	Sequence alignment
Ncam1	'Lower'	P13596 (IPI00476991)	^{12}FFLGTAVSL..........^{349}ISSEEKASWTRPEKQETLD..........^{648}KYRA-LAS
	'Higher'	F1LUV9 (IPI00777130)	^{1}FFFSFAVSL..........^{338}ISSEEK - - - - - - - - - - TLD..........^{627}KYRAKLAS
Strbp	'Lower'	Q6XD99 (IPI00555287)	^{48}LADER..........^{1410}KDLTSV—NILL<br>KKQQ--MLENQMEVRKKEIEELQSQAQALSQEGKSTDEVDSK
	'Higher'	Q5D002 (IPI00952273)	^{1}LTDER..........^{1363}KDLTTSAQNKVL<br>DGASVFLLMYSCPPRCWRIRWKRGRAQMR- - - - - - - - - -DSK
Sptbn1	'Lower'	Q9JKU6 (IPI00952472)	^{509}GPILTAWQKS-CDGANEKRR..........^{554}GPNKKVAK -- QVQRALEKLF
	'Higher'	Q9JKU6 (IPI00327397)	^{509}GPILTASGKNPVMELNEKRR..........^{555}GPNKKVAKASAALAALEKLF
Wdr47	'Lower'	G3V9M3 (IPI00768998)	^{381}VETQQP-AFEPMCQGSGLEK
	'Higher'	Q5BJR0 (IPI00949240)	^{381}VETQQPVSIEPMCQGSGLEK
